# Erratum

**DOI:** 10.1200/JOP.2016.019638

**Published:** 2016-12

The April 2016 article by Balch et al, entitled, “The National Practice Benchmark for Oncology: 2015 Report for 2014 Data,” (J Oncol Pract 12:e437-e475, 2016) contained an error.

The following acknowledgment was not included in this publication: **The production of this manuscript was funded by the Conquer Cancer Foundation Mission Endowment.**

This has been corrected as of November 14, 2016.

